# Mortality and Outcomes of Pediatric Tracheostomy Dependent Patients

**DOI:** 10.3389/fped.2021.661512

**Published:** 2021-05-04

**Authors:** Kiran B. Hebbar, Ajay S. Kasi, Monica Vielkind, Courtney E. McCracken, Caroline C. Ivie, Kara K. Prickett, Dawn M. Simon

**Affiliations:** ^1^Division of Pediatric Critical Care Medicine, Children's Healthcare of Atlanta, Emory University, Atlanta, GA, United States; ^2^Division of Pediatric Pulmonology, Children's Healthcare of Atlanta, Emory University, Atlanta, GA, United States; ^3^Pediatric Biostatistics Core, Children's Healthcare of Atlanta, Emory University, Atlanta, GA, United States; ^4^Division of Pediatric Otolaryngology, Children's Healthcare of Atlanta, Emory University, Atlanta, GA, United States

**Keywords:** tracheostomy, pediatric, outcome, mortality, decannulation

## Abstract

**Objective:** To describe clinical factors associated with mortality and causes of death in tracheostomy-dependent (TD) children.

**Methods:** A retrospective study of patients with a new or established tracheostomy requiring hospitalization at a large tertiary children's hospital between 2009 and 2015 was conducted. Patient groups were developed based on indication for tracheostomy: pulmonary, anatomic/airway obstruction, and neurologic causes. The outcome measures were overall mortality rate, mortality risk factors, and causes of death.

**Results:** A total of 187 patients were identified as TD with complete data available for 164 patients. Primary indications for tracheostomy included pulmonary (40%), anatomic/airway obstruction (36%), and neurologic (24%). The median age at tracheostomy and duration of follow up were 6.6 months (IQR 3.5–19.5 months) and 23.8 months (IQR 9.9–46.7 months), respectively. Overall, 45 (27%) patients died during the study period and the median time to death following tracheostomy was 9.8 months (IQR 6.1–29.7 months). Overall survival at 1- and 5-years following tracheostomy was 83% (95% CI: 76–88%) and 68% (95% CI: 57–76%), respectively. There was no significant difference in mortality based on indication for tracheostomy (*p* = 0.35), however pulmonary indication for tracheostomy was associated with a shorter time to death (HR: 1.9; 95% CI: 1.04–3.4; *p* = 0.04). Among the co-morbid medical conditions, children with seizure disorder had higher mortality (*p* = 0.04).

**Conclusion:** In this study, TD children had a high mortality rate with no significant difference in mortality based on indication for tracheostomy. Pulmonary indication for tracheostomy was associated with a shorter time to death and neurologic indication was associated with lower decannulation rates.

## Introduction

Advances in pediatric critical care medicine have increased survival of complex medical patients for whom tracheostomy and/or home mechanical ventilation is required (1, 2). Indications for tracheostomy have broadened over time and include more chronic medical conditions such as neurologic disease and chronic respiratory failure requiring home mechanical ventilation (3–5). These chronic conditions pose challenges to clinicians when counseling families of the potential benefits and outcomes after tracheostomy (3, 6). Although pediatric tracheostomy rates have decreased, tracheostomy dependent (TD) patients have become increasingly complex, placing a greater burden on our healthcare system and the families caring for them (2, 3).

Mortality estimates of TD children are variable and range from 13 to 20% (7–16). Although several studies have reported risk factors associated with increased mortality and decannulation outcomes in TD children, almost 50% of deaths were unexpected (5–7, 11, 17). Therefore, we conducted a retrospective review of our cohort of TD children to describe their mortality and risk factors associated with mortality. The objectives of this study were to: (1) identify the mortality rate, (2) identify risk factors that are associated with increased mortality, and (3) describe the causes of mortality in our TD cohort.

## Materials and Methods

The study is a retrospective review of patients with new and established tracheostomies hospitalized at Children's Healthcare of Atlanta at Egleston (Children's) between January 2009 and December 2015. Children's is a 272-bed pediatric hospital with a 45-bed neonatal intensive care unit, 36-bed pediatric intensive care unit, 27-bed cardiac intensive care unit, and an 11-bed technology-dependent intensive care unit for TD patients. Approximately 30 tracheostomies are performed annually. The study was approved by the Institutional Review Board at Children's.

Hospitalized patients with tracheostomy 0–18 years of age were included. Patients were identified and the data for each patient was collected manually by chart review of hospitalized patients during the study period. Patients were excluded if there was no information pertaining to tracheostomy placement or if the patient were transferred to another facility during the study period. De-identified demographic and clinical information were collected for patients hospitalized during the study duration until death, tracheostomy decannulation, or August 2016 (whichever occurred last). The recorded data included, but were not limited to, age at tracheostomy, indication for tracheostomy, co-morbid medical conditions, number and duration of readmissions, date of death or decannulation, and cause of death, if known. In this study, we defined TD patient as any patient with a tracheostomy during the study period.

Patients were categorized into three groups by two independent authors based on their primary indication for tracheostomy—(1) pulmonary, (2) anatomic/airway obstruction, and (3) neurologic causes. When a patient had more than one indication for tracheostomy (e.g., chronic lung disease and anatomic/airway obstruction), two independent authors determined a primary indication after thoroughly reviewing the patient's clinical information.

### Outcome Measures

The outcome measures were overall mortality rate, mortality risk factors, and causes of death. Mortality was further characterized as time to death following tracheostomy, location, and cause of death (if known). The mortality rate was analyzed based on indication for tracheostomy and the presence of co-morbid medical conditions to determine the risk factors contributing to mortality. Secondary outcomes included time to decannulation following tracheostomy, and hospital use—duration of hospitalization following tracheostomy, readmissions related to any cause at Children's, and length of stay per hospitalization.

### Statistical Analysis

Descriptive statistics were calculated for all variables of interest and include counts and percentages or medians and ranges, as appropriate. Baseline demographic and clinical characteristics at the time of tracheostomy were compared between survivors and non-survivors using Chi-square tests for categorical variables and Wilcoxon rank-sum tests for continuous variables. Overall survival and time to decannulation were treated as time dependent outcomes and were analyzed using survival analysis methods. Distribution of survival times was plotted using a Kaplan-Meier curve and risk factors associated with death following tracheostomy were identified using Cox proportional hazard regression. The proportional hazard assumption was verified by testing the weighted Schoenfeld residuals. Risk factors associated with death are presented as hazard ratios (HR) with associated 95% confidence intervals (CI). The Fine and Gray method was used to estimate the cumulative incidence of decannulation while accounting for death as a competing event. Analyses were conducted using SAS v 9.4 (Cary, NC) and statistical significance was assessed at the 0.05 level, unless otherwise noted.

## Results

A total of 187 patients were identified during the study period. Twenty-three patients (12%) were excluded because of incomplete information or loss to follow-up. The demographic information of our cohort is summarized in Table 1. The median age at tracheostomy was 6.6 months (IQR 3.5–19.5 months). The median duration of follow-up was 23.8 months (IQR 9.9–46.7 months) and the longest duration of follow-up was 181.5 months. The indications for tracheostomy and co-morbid medical conditions are described in Table 2.

**Table 1:** Demographics and outcomes of pediatric tracheostomy cohort.

**Variables** ** (*N* = 164, unless otherwise noted)**	**Overall** ***N* = 164**	**Alive at follow-up** ** (*N* = 119)**	**Died** ** (*N* = 45)**	***P*-value**
Sex				0.299
Female	69 (42.1%)	53 (44.5%)	16 (35.6%)	
Male	95 (58.7%)	66 (55.5%)	29 (64.4%)	
Race/ethnicity				0.505
Caucasian	54 (32.9%)	39 (32.8%)	15 (33.3%)	
African American	93 (56.7%)	68 (57.1%)	25 (56.6%)	
Hispanic	12 (7.3%)	8 (6.7%)	4 (8.9%)	
Indian	2 (1.2%)	2 (1.7%)	0 (0%)	
Unknown	1 (0.6%)	0 (0%)	1 (2.2%)	
Asian	2 (1.2%)	2 (1.7%)	0 (0%)	
Gestational age (weeks) (*n* = 159) Median (25–75th)	37 (27–39)	37 (27–39)	36.5 (29.5–39)	0.838
Birth weight (grams) (*n* = 160) Median (25–75th)	2,483 (1,033–3,715)	2,509 (1,047–3,144)	2,483 (951–3,370)	0.671
Weight at tracheostomy (grams) (*n* = 149) Median (25–75th)	5,800 (4,240–9,000)	5,600 (4,005–9,700)	6,250 (4,640–8,100)	0.920
Age at tracheostomy (months) Median (25–75th)	6.61 (3.45–19.46)	6.58 (3.48–21.4)	6.64 (3.42–10.09)	0.694

**Table 2:** Clinical characteristics and outcomes.

**Variables**	**Overall** ***N* = 164**	**Alive at follow-up** ** (*N* = 119)**	**Died** ** (*N* = 45)**	***P*-value**
**Primary indication for tracheostomy**^**a**^				
Pulmonary	66 (40.2%)	44 (37.0%)	22 (48.9%)	0.165
Neurologic	39 (23.8%)	29 (24.4%)	10 (25.6%)	0.773
Anatomic/airway obstruction	59 (36.0%)	46 (38.7%)	13 (28.9%)	0.245
**Comorbid medical conditions**				
Congenital heart disease^b^				0.277
None	105 (64.0%)	80 (67.2%)	25 (55.6%)	
Simple	42 (25.6%)	29 (24.3%)	13 (28.9%)	
Complex	17 (10.4%)	10 (8.4%)	7 (15.6%)	
Pulmonary hypertension^c^	39 (23.8%)	27 (22.7%)	12 (26.7%)	0.593
Seizure disorder	34 (20.7%)	20 (16.8%)	14 (31.1%)	0.044^*^
Structural CNS anomaly	38 (23.2%)	23 (19.3%)	15 (33.3%)	0.058
Feeding issues	153 (93.3%)	112 (94.1%)	41 (91.1%)	0.729
Genetic condition	49 (29.9%)	31 (26.1%)	18 (40.0%)	0.082
Other^d^	14 (8.5%)	9 (7.6%)	5 (11.1%)	0.533

*CNS, Central nervous system*.

* *Statistically significant*.

a *Examples of pulmonary etiologies included chronic respiratory failure from bronchopulmonary dysplasia, pulmonary hypoplasia, congenital diaphragmatic hernia, and failure to extubate requiring prolonged assisted ventilation. Anatomical airway indications included subglottic stenosis, airway malacia, vocal cord paralysis, craniofacial disorders, and airway trauma. Neurologic indications included chronic respiratory failure from neuromuscular diseases (e.g., spinal muscular atrophy, Duchenne muscular dystrophy), traumatic brain/spinal cord injury, brainstem tumors, and hypoventilation syndromes*.

b *Simple congenital heart disease included single intracardiac shunts such as atrial or ventricular septal defect and patent ductus arteriosus. Complex congenital heart disease included major structural cardiovascular malformations such as tetralogy of Fallot, total or partial anomalous pulmonary venous return, and hypoplastic left heart syndrome*.

c *Pulmonary hypertension was diagnosed based on indirect evidence on echocardiography*.

d *Other- examples are hematologic/oncologic conditions (leukemia, retinoblastoma, anemia, myelodysplastic syndrome), gastrointestinal conditions (omphalocele, Crohn's disease, gastroschisis), and endocrine conditions (diabetes insipidus)*.

### Patient Survival and Mortality

**Figure 1:**
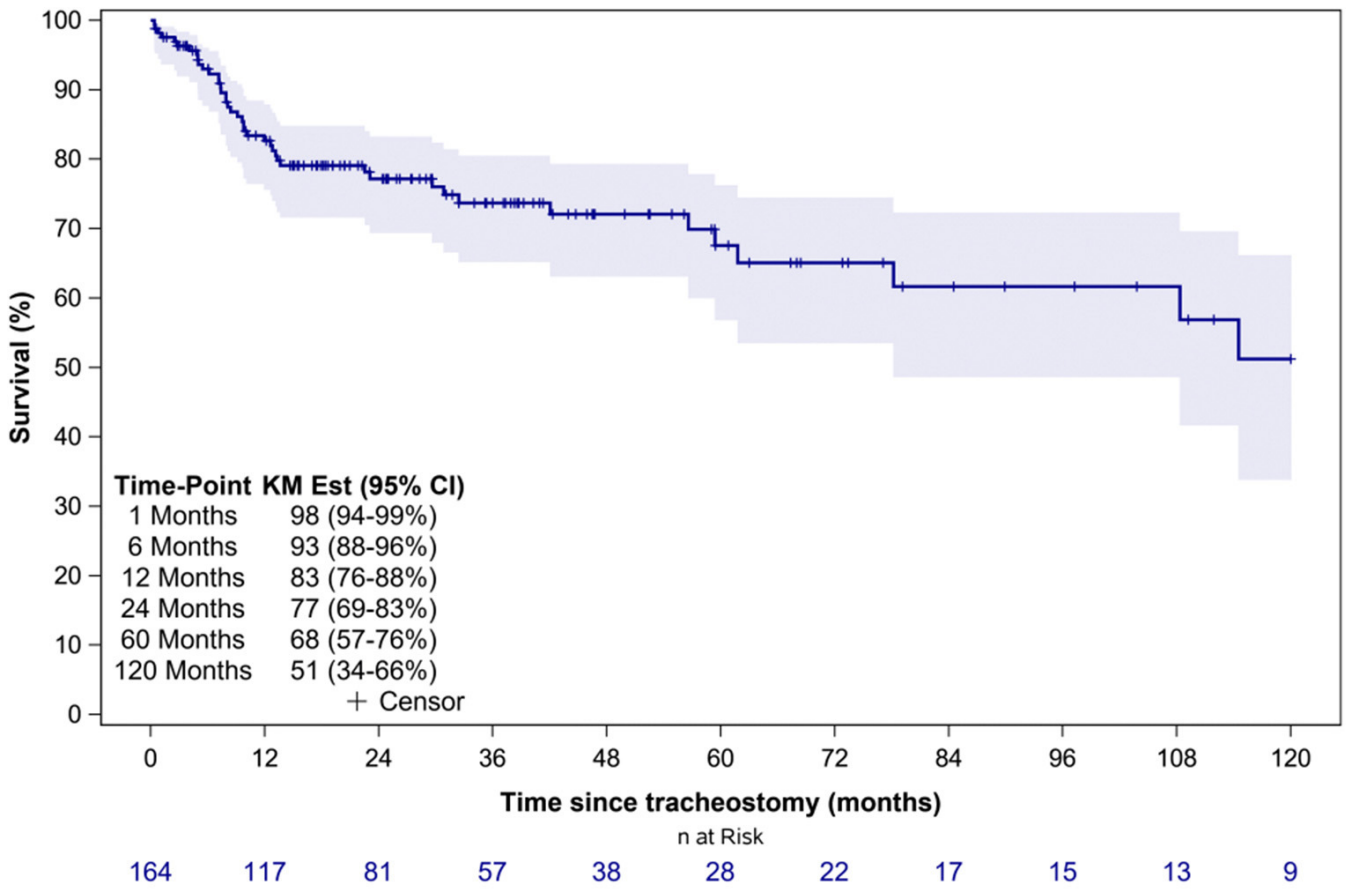
Overall survival estimates of the entire cohort. KM Est, Kaplan-Meier survival estimates; CI, confidence interval.

**Figure 2:**
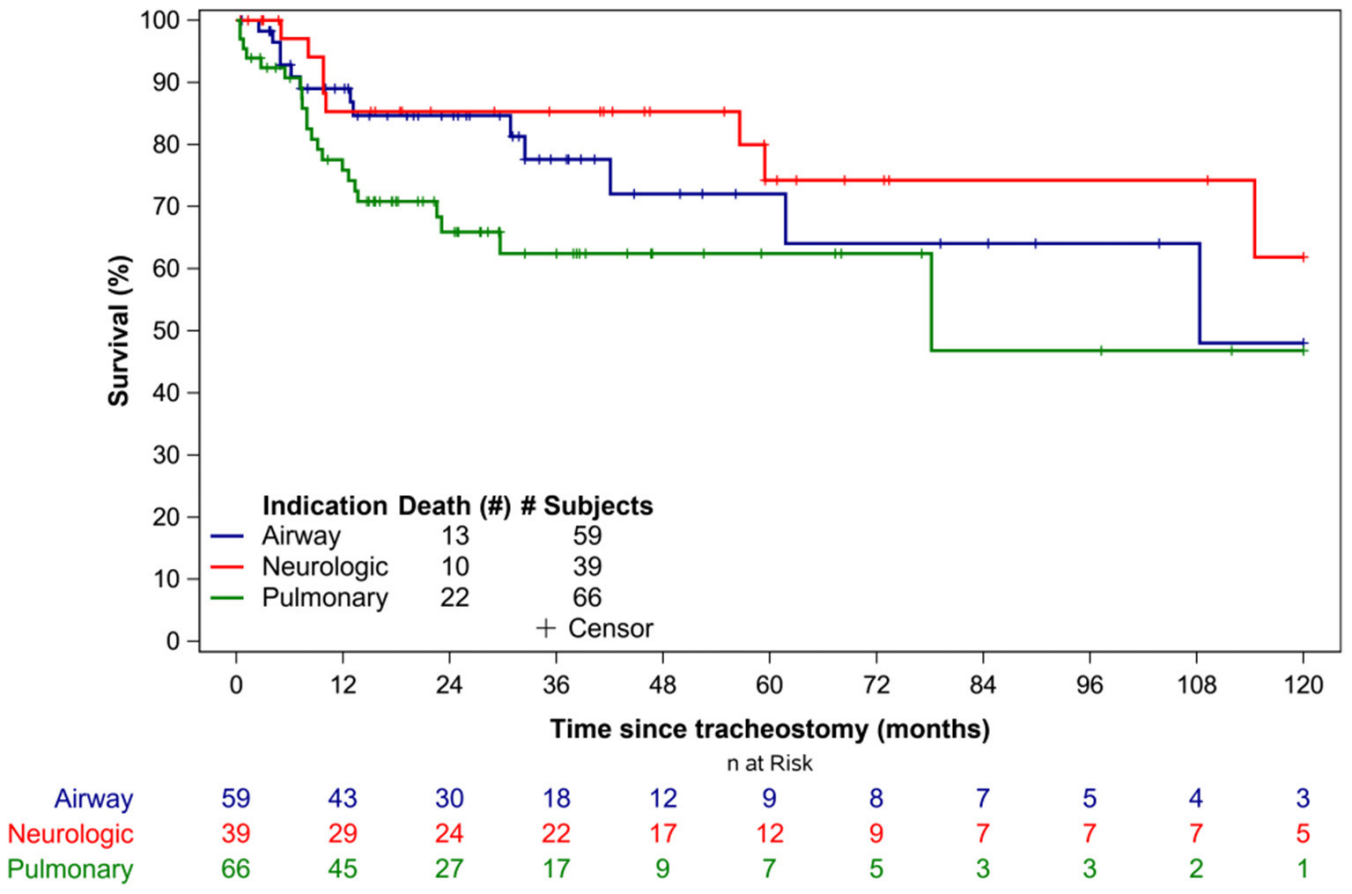
Survival estimates based on the indication for tracheostomy.

Overall, 45 patients (27%) died during the study period. The median time to death following tracheostomy was 9.8 months (IQR 6.1–29.7 months) (Table 3). Figure 1 illustrates the Kaplan-Meier survival curves for the cohort. Figure 2 illustrates the survival estimates based on the indication for tracheostomy. When stratified by tracheostomy indication, mortality was highest in the pulmonary group with 22 deaths (49%) (Table 2). There was no statistically significant difference in the mortality among the 3 groups (*p* = 0.35). Among the co-morbid medical conditions, children with seizure disorder had higher mortality (*p* = 0.04). Fifty-nine patients (36% of the cohort) had congenital heart disease (CHD) that contributed to 20 (44%) deaths. Of the 66 patients in the pulmonary group, 29 also had CHD. Among patients in the pulmonary group, the mortality was 38% (11/29 patients) with CHD compared to 30% (11/37 patients) without CHD (*p* = 0.48). Pulmonary disease was the only significant risk factor associated with time to death (*p* = 0.04) (Table 4).

**Table 3:** Outcomes and hospital utilization.

**Outcome** ** (*N* = 164, unless otherwise noted)**	**N (%), unless** ** otherwise noted**
Death	45 (27.4%)
Median time (days) (25–75th) from tracheostomy to death (*n* = 45)	298 (187–904)
Decannulation	36 (22.0%)
Median time (days) (25–75th) from tracheostomy to decannulation (*n* = 36)	744 (305–1,098)
Length of stay after tracheostomy, median (25–75th)	51 (34–77)
Time from discharge to first readmission (*n* = 137), median (25–75th)	44 (14–159)
Total hospitalizations following tracheostomy, median (25–75th)	3 (1–7)
Total days in the ICU following discharge from tracheostomy admission, median (25–75th)	16 (2–55)
Number of outpatient procedures, median (25–75th)	0 (0–1)

*ICU, pediatric intensive care unit*.

**Table 4:** Analysis of risk factors associated with mortality.

**Variables**	**Hazard ratio 95% CI**	***P*-value**
**Sex**		
Female	0.72 (0.39–1.33)	0.293
Male (ref)	–	
Gestational age (weeks) (*n* = 158) (per week)	1.01 (0.96–1.06)	0.765
Weight at tracheostomy (grams) (*n* = 148) (per 1,000 grams)	1.00 (0.98–1.02)	0.767
Age at tracheostomy (per month)	1.00 (0.99–1.01)	0.766
**Primary indication for tracheostomy**		
Pulmonary	1.89 (1.04–3.43)	0.037^*^
Neurologic	0.66 (0.32–1.34)	0.249
Anatomic/airway obstruction	0.73 (0.38–1.38)	0.329
**Comorbidities**		
Congenital heart disease		
None (ref)	–	
Simple	1.33 (0.68–2.60)	0.407
Complex	1.72 (0.73–4.02)	0.213
Pulmonary hypertension	1.16 (0.60–2.25)	0.661
Seizure disorder	1.24 (0.65–2.36)	0.512
Structural CNS anomaly	1.51 (0.81–2.82)	0.195
Feeding issues	0.58 (0.21–1.63)	0.299
Genetic condition	1.33 (0.73–2.43)	0.354
Other^a^	1.11 (0.44–2.81)	0.833

*CI, Confidence interval; CNS, Central nervous system*.

* *Statistically significant*.

a *Other- examples are hematologic/oncologic conditions (leukemia, retinoblastoma, anemia, myelodysplastic syndrome), gastrointestinal conditions (omphalocele, Crohn's disease, gastroschisis), and endocrine conditions (diabetes insipidus)*.

### Analysis of Mortality Events

Twelve deaths (27%) were due to tracheostomy-related accidents (TRA) that included accidental decannulation, hemorrhage from tracheostomy, and mucus plugging (Table 5). Five deaths were due to progression of underlying disease, 2 of which involved withdrawal of support. Of the 3 patients who died from cardiac arrest, 2 had CHD (tetralogy of Fallot and hypoplastic left heart syndrome). For 16 (36%) patients, cause of death remained unexplained or unknown. Thirty-four deaths (76%) occurred in the outpatient setting and 11 deaths (24%) occurred in the hospital of which six deaths occurred during the initial hospitalization for tracheostomy placement.

**Table 5:** Cause of death (*N* = 45).

**Cause of death**	**N (%)**
Unexplained/unknown	16 (35.5%)
Tracheostomy accidents	12 (26.6%)
Acute respiratory failure	5 (11.1%)
Progression of underlying disease	5 (11.1%)
Cardiac arrest	3 (6.6%)
Shock—septic and hypovolemic	2 (4.4%)
Pulmonary hypertension	2 (4.4%)

### Tracheostomy Decannulation

**Figure 3:**
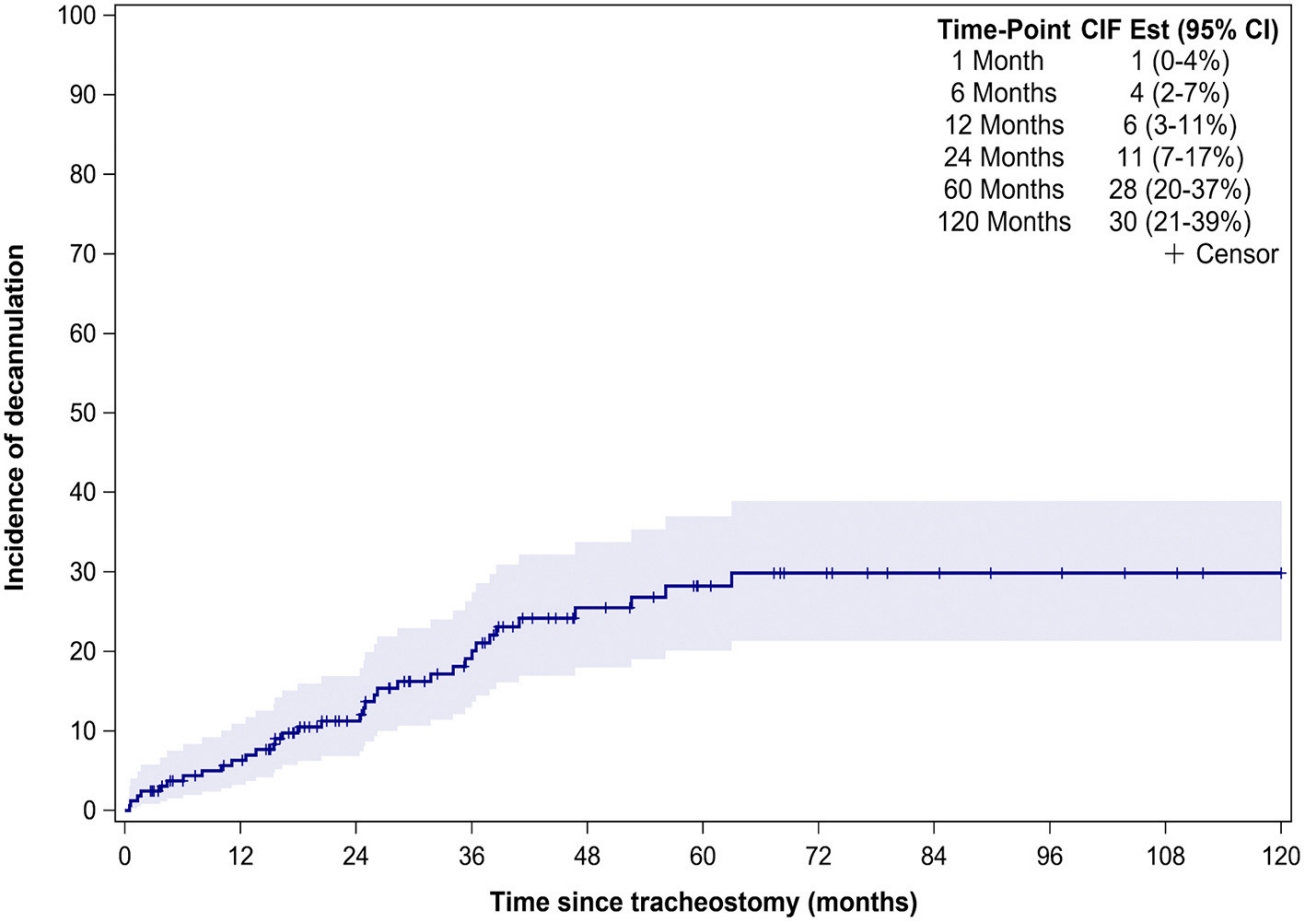
Time from tracheostomy to decannulation for the entire cohort. CIF Est, Cumulative incidence function estimates; CI, confidence interval.

**Figure 4:**
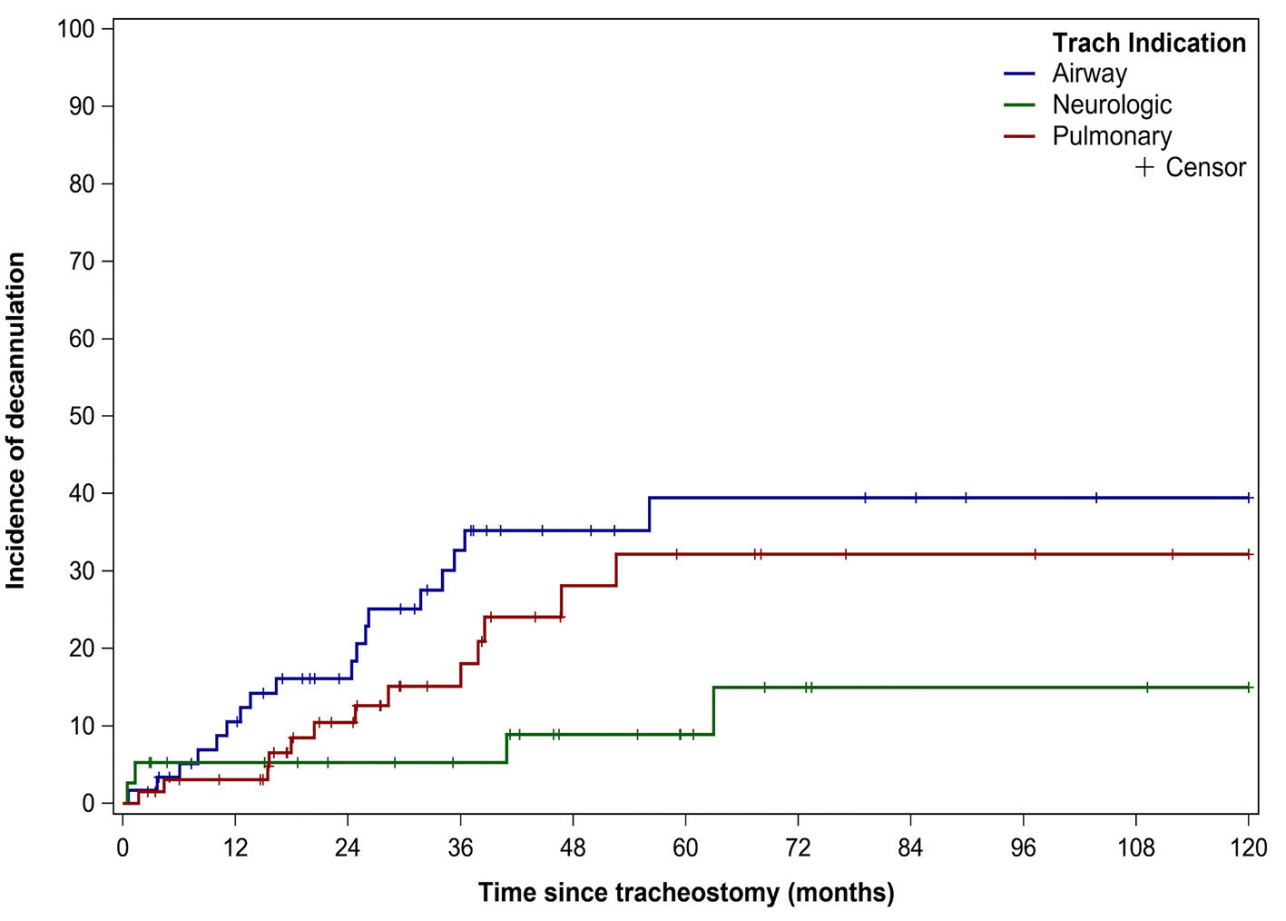
Decannulation based on indication for tracheostomy. Decannulation rates based on tracheostomy indication: airway obstruction (18/59, 31%), pulmonary (13/66, 20%), and neurologic (5/39,13%). Trach, Tracheostomy.

Thirty-six (22%) patients underwent tracheostomy decannulation. Figure 3 illustrates the cumulative incidence curve for time to decannulation following tracheostomy. The median time to decannulation following tracheostomy was 24.5 months (IQR 10–36.1 months). There was no difference in decannulation rates based on tracheostomy indication (*p* = 0.1) (Figure 4). Among patients in the pulmonary group, there was no difference in decannulation for those with or without CHD (*p* = 0.29). When controlling for death as a competing event, time to event analysis showed that patients in the airway group were more likely to be decannulated compared to patients in the neurologic group (HR = 3.63, 95% CI: 1.23–10.66, *p* = 0.02).

Hospital utilization is described in Table 3. The duration of hospitalization was not significantly different between survivors and non-survivors during the study period (*p* = 0.22).

## Discussion

This study evaluated the clinical characteristics and outcome of a large cohort of TD children and adds to the growing literature regarding mortality and outcomes after tracheostomy. Overall, we found a high mortality in our TD patients, a high mortality from TRA, and a low probability of decannulation after 5 years of tracheostomy. Additionally, patients with a pulmonary indication for tracheostomy had an earlier time to death.

The overall mortality (27%) is higher than mortality rates (13–23%) in other retrospective studies of TD children (6–16). Similar to other studies, we report higher mortality in children with cardiopulmonary disease (7, 18). Funamura et al. reported the mortality in pediatric TD patients varied by the indication for tracheostomy, with cardiopulmonary disease being the most common indication (35%) accounting for 27% of mortality and increased risk of death (7). McPherson ML et al. also reported that mortality varied by the indication and a respiratory etiology accounted for 25% of tracheostomy (6). However, pulmonary disease independent of cardiac disease, accounted for 40% of our cohort. The high mortality rate in our study may be attributed to the large pulmonary group that accounted for almost half of all deaths. Although gestational age was not a significant risk factor for mortality in our study, a nationwide database study showed that prematurity was associated with higher mortality following tracheostomy (3).

Unlike prior reports, weight and age at tracheostomy were not significant risk factors for death, which was surprising given the suspected higher risk of plugging anticipated in smaller tracheostomy tubes (3). Despite 45% of TD children with CHD not surviving, CHD and pulmonary hypertension were not significant risk factors associated with time to death. Nonetheless this striking mortality rate is consistent with prior reports and should be considered in pre-operative counseling prior to tracheostomy (3, 7, 19, 20). Edwards et al. reported that children with complex CHD and tracheostomy-ventilator dependence had higher mortality rates and less success weaning off mechanical ventilation (20). In our study, even when TD children were stratified as simple or complex CHD, no difference in mortality was noted between the groups. Co-morbidities in the TD child with CHD such as chromosomal abnormalities, cardiac dysfunction, structural airway abnormalities, and phrenic nerve injury may be contributing factors for increased mortality and failure to wean from mechanical ventilation (19).

Previous studies have reported decannulation rates between 17 and 75% in TD children, compared to 22% in our study (6, 8–10, 13–15, 21). This variation in decannulation rates are likely due to the indication for tracheostomy (6, 8, 11). TD children are a heterogenous population with tracheostomy performed for a variety of indications that are also institution and practice dependent. Lower decannulation rates have been reported in TD children with cardiopulmonary and neurologic disease (6, 8). At 5.5 years of follow up, only 15% of patients in the neurologic group were decannulated (Figure 3). The lower decannulation rate in our study may be attributed to the large pulmonary and neurologic groups (64% of our cohort) and the heterogeneous composition of TD patients in prior studies. The low probability of decannulation beyond 5 years and in children with neurologic disease would be important for families being counseled on tracheostomy.

The median time to decannulation (2 years) in our study is in the previously reported range of 0.3–5 years (6, 8–11, 13, 15, 16). This wide range in time to decannulation may be attributed to institution-dependent clinical practices and the indication for tracheostomy. Early tracheostomy decannulation, when feasible, may reduce mortality related to tracheostomy-related causes which has been reported to be up to 19% (17).

Our study shows that the overall survival of TD children declines over time (Figure 1). The proportion of surviving children declined by 10% between 6 months and 1 year of tracheostomy. Several factors may influence this increased mortality in the 1st year of tracheostomy including progression of underlying disease, co-morbid medical conditions, TRA, and reduced outpatient resources in contrast to inpatient management. Progression of underlying disease and co-morbid medical conditions may be inherent characteristics of each patient, however mortality related to TRA are potentially avoidable causes of death (17). In our study, mortality due to TRA was higher compared to other studies which report mortality rates of 0.7–19% (7, 9, 10, 13, 14, 16, 17). The higher overall mortality is likely related to the larger pulmonary group accounting for almost half of all deaths, shortage of skilled home healthcare nursing (HHN) in our region, and reduced outpatient resources for families. Most deaths occurred in the outpatient setting where families have seen a reduction in HHN and a rise in patient complexity.

Although we did not characterize cause of death based on duration of tracheostomy, there was increased risk of death in the 1st year following tracheostomy. This may reflect difficulties with both stabilization of the medical conditions leading to tracheostomy and challenges with transitioning from hospital to home-based care. At our institution, caregiver education and transitioning to home confers with current guidelines where caregivers demonstrate home care skills and HHN is arranged prior to discharge (22, 23). Because most pediatric TD patients are medically complex and often qualify as disabled, funding for HHN is typically received through state-sponsored insurance, leading to interstate variability in the number of staffed nursing hours per patient. Despite HHN approval, a shortage of staffed nursing is prevalent in our region (anecdotal) and we often receive parental reports of understaffed HHN and inexperienced nurses especially in the rural parts of our state. In addition, studies have reported a deficit in the knowledge of primary caregivers and HHN in the management of tracheostomy and ventilator-dependent children (17, 24–26). On review of the literature, there is a lack of industry recognized best practices or core clinical competencies in HHN training in tracheostomy care. Periodically assessing caregiver knowledge and skills in addition to training HHN in managing tracheostomy emergencies may help reduce preventable causes of death in TD children. With understaffed HHN, caregivers are imposed with the responsibility of caring for their TD child. This drop-off in outpatient resources when compared to inpatient care may impact families and contribute to the high tracheostomy-related mortality seen in our study. The cause of death could not be fully explained or was unknown in 16 patients. Unexplained or unexpected deaths are reported in children with chronic conditions and have been frequently attributed to their underlying co-morbid medical conditions (17).

TD children often have multiple co-morbid medical conditions that may influence their length of stay and readmission rates. Our data showed that TD children experience prolonged hospitalizations, frequent readmissions, and spend many days in the intensive care unit in subsequent readmissions consistent with other studies (2, 4–6, 11, 12, 18). This information would aid clinicians to set expectations for families based on tracheostomy indications, comorbidities, high resource utilization, and care coordination prior to tracheostomy.

Our study is limited by the single institution retrospective design. Since we are a regional referral center for medically complex children, our results may be subject to a referral bias and skewed with more medically complex patients that may explain the higher mortality rate in our cohort. Future studies that attempt to stratify severity of pulmonary disease and other comorbidities may provide more generalizable results. Although patients excluded from our analysis due to incomplete information or loss to follow-up may have affected the overall results, we believe they do not differ significantly with those patients included in our study. Data on hospitalizations, length of stay, and procedures were limited to hospitalizations only at Children's. The primary indication for tracheostomy was determined by the authors and patients were not stratified into subgroups with several comorbidities necessitating the tracheostomy. In addition, patients were not stratified based on severity using mortality risk indices and there was lack of reported modeling to assess risk for covariates. Patients included toward the end of the study period may not have follow up data to assess 1-and 5-year mortality. Lastly, given the retrospective study design, outpatient deaths or circumstances leading to death are limited to existing data in the medical records and may be underreported if patients moved out-of-state or were lost to follow up.

## Conclusion

Despite advances in pediatric critical care, TD children continue to have a high mortality rate with an increased risk of death in the 1st year after tracheostomy. The presence of underlying seizure disorder was associated with higher mortality. More granular outpatient and in-home data is required to better identify target etiologies for mortality in this growing patient population.

## Data Availability Statement

The original contributions presented in the study are included in the article/supplementary material, further inquiries can be directed to the corresponding author/s.

## Ethics Statement

The studies involving human participants were reviewed and approved by Children's Healthcare of Atlanta Institutional Review Board. Written informed consent from the participants' legal guardian/next of kin was not required to participate in this study in accordance with the national legislation and the institutional requirements.

## Author Contributions

KH, MV, KP, and DS contributed to conception and design of the study. MV and CI collected and organized the data. CM performed the statistical analysis and designed the figures. AK wrote the first draft of the manuscript. KH, DS, KP, and CM wrote sections of the manuscript. All authors contributed to manuscript revision, read, and approved the submitted version.

## Conflict of Interest

The authors declare that the research was conducted in the absence of any commercial or financial relationships that could be construed as a potential conflict of interest.

## Abbreviations

CHD: congenital heart disease
CI: confidence interval
HHN: home healthcare nursing
HR: hazard ratio
IQR: interquartile range
TD: tracheostomy dependent
TRA: tracheostomy-related accidents.
